# Biological control of *Meloidogyne* spp. in glasshouse-grown chrysanthemum

**DOI:** 10.21307/jofnem-2020-125

**Published:** 2021-01-09

**Authors:** J. R. De Long, M. A. Streminska, A. Persijn, H. M. I. Huisman, C. van der Salm

**Affiliations:** Wageningen University & Research BU Greenhouse Horticulture & Flower Bulbs

**Keywords:** Biological control, Biostimulant, Chrysanthemum, Glasshouse, Host-parasitic relationship, *Meloidogyne*, Root-knot nematode, Resistance, Sustainable horticulture

## Abstract

Root-knot nematodes (*Meloidogyne* spp.) are a major problem in soil-based glasshouse-grown chrysanthemums. To combat root-knot nematodes in the glasshouse, the soil is typically steamed every 5–6 production cycles. However, this method is expensive, environmentally unfriendly and reduces resistance and resilience of the soil against pathogens and pests. Here, we added biological pesticides/a basic substance and biostimulants both individually and in combination to determine individual or interactive effects against damage by root-knot nematodes in chrysanthemums. We found that the application of biological nematicides derived from garlic extract, the basic substance chitosan HCl and biostimulants comprised of sea minerals and plant oils correlated with reduced root-knot nematode damage. These effects may have been due to direct effects against the nematodes or through indirect effects such as increased resistance and resilience of the plants. Overall, the biostimulants increased the total number of free-living nematodes in the soil, which could lead to a beneficial increase in nutrient cycling in the soils. Our results demonstrate that biological reagents show promise in reducing root-knot nematode damage in glasshouse-grown chrysanthemum and may lead to more resistance and resilient soils.

Root-knot nematodes (RKN; *Meloidogyne* spp.) are a worldwide problem in the cultivation of both fruit and vegetable crops (Jones et al., 2013). There are approximately 100 different species of *Meloidogyne* (with new species being described often) (Jones et al., 2013) and specific species or “races” usually parasitize only a select range of host plants (Moens et al., 2009). The life cycle of RKN lasts approximately 30 days, depending on species and environmental conditions (Ploeg and Maris, 1999). Each female lays 200–500 eggs on the surface of the roots or inside the gall tissue. Once the eggs hatch, the stage 2 (J2) juvenile penetrates the roots of a suitable host plant and induces the formation of a “giant cell,” from which the nematode obtains carbohydrates. The hyper-division of root cells that occurs during the creation of the giant cells restricts water and nutrient flow between above- and belowground compartments, thereby leading to damage and possible death of the host plant.

The species *M. incognita* and *M. javanica* are two of the most economically damaging pests in glasshouse-grown chrysanthemums that are produced for the cut flower industry in the Netherlands (Amsing, 2003, 2004). To combat RKN, chemical nematicides such as oxamyl are used (Desaeger et al., 2004). More commonly, the glasshouse soil is steamed every 5–6 growing cycles (*c*. once per year). Steaming is capable of suppressing various other soil-bound diseases such as Pythium, Fusarium, Rhizoctonia, Verticillium and thrips (pupae) (Raaphorst, 2010). However, this method uses a lot of energy: 3.5 m^3^/m^2^ gas per round of steaming, which, based on 350 ha of chrysanthemum cultivation in the Netherlands, amounts to *c*. 14 Mm^3^ gas per year (van der Wurff et al., 2009), leading to the emission of a substantial amount of greenhouse gases. Taken collectively, steaming is expensive, environmentally unfriendly and lowers the resistance and resilience of the soil against other pathogens and pests (Bollen, 1974; Vatchev, 2015). Further, the use of chemical nematicides is being phased out due to their unwanted environmental side effects (Wesemael et al., 2011; Donley, 2019). Therefore, it is necessary to find environmentally friendly and cost-effective options to combat RKN.

Numerous alternative strategies are being developed against RKN that involve amendments to the soil (Oka, 2010). Biological nematicides that use fungi antagonistic to the nematodes (e.g., *Purpureocillium lilacinum* strain 251) have had success in tomato cultivation (Kiewnick and Sikora, 2006; Dahlin et al., 2019) and products with this fungi are already on the market (e.g., BioAct Prime from Bayer). However, products with this fungi have had limited success in combating RKN in chrysanthemum (van der Wurff, 2010). Some isolates of bacteria such as *Bacillus* spp. (Oka et al., 1993) and *Pseudomonas* spp. (Kloepper et al., 1992) have also shown success in control root-knot nematodes, again typically in tomatoes. Extracts derived from garlic (*Allium sativa*) have shown promising effects against *Meloidogyne* spp. juveniles in the soil and reduced root-knot formation during cucumber cultivation (Al-Shalaby, 2009). Further, adding so-called “basic substances” (www.ctgb.nl/onderwerpen/basisstoffen date queried: 10.06.2020), such as those containing chitosan HCl, to the soil has been shown to reduce RKNs due to toxic levels of ammonia mineralization in the soil (Spiegel et al., 1987) and through the promotion of microorganisms that produce chitinases, which dissolve the shells of nematode eggs and inhibits the molting of adults (Akhtar and Alam, 1993; Chen and Peng, 2019). Nonetheless, there remains a dearth of knowledge as to which biological nematicides and basic substances are as or more effective than conventional methods such as steaming to reduce RKN damage in chrysanthemum.

Progress has also been made in the development of so-called biostimulants. The EU defines biostimulants as substances that can increase the resistance/resilience of a plant to abiotic stress (https://biostimulants.eu/), while fundamental research broadens this definition to include biotic stresses (Bulgari et al., 2015; Ricci et al., 2019). These substances can increase the resistance and resilience of plants indirectly by increasing beneficial organisms in the soil (Bulgari et al., 2015) and by direct effects, such as by increasing secondary defense compounds and/or other metabolites in the plants (Goñi et al., 2018; Parađiković et al., 2019) or replenishing micronutrients, as is the case with sea mineral extracts (Petralia, 2016; Zaseybida and Wilkes, 2016). Biostimulants derived from plant oils have shown promising effects against RKN (Laquale et al., 2015) and in increasing general resilience in plants (Khan et al., 2009). Further, the waste products of insect rearing can also act as biostimulants due to the effects of the chitin contained within the exoskeletons of insects (same mechanisms as described above) (Pichyangkura and Chadchawan, 2015). Combined effects of the addition of both biological and chemical nematicides alongside biostimulants have been seen in the combat of RKN in tomatoes (Dahlin et al., 2019). Even though evidence suggests positive effects of both biological nematicides and biostimulants against RKN in tomatoes and also possible interactions between these substances (Liu et al., 2014; Yakhin et al., 2017), much less is known in other crops such as chrysanthemum. It also remains unknown whether there are interactions between biological pesticides and biostimulants that might have additional benefits for chrysanthemums.

In this experiment, we tested the individual and combined effects of a chemical nematicide, a biological nematicide and a basic substance, as well as biostimulants, on *Meloidogyne* spp. and the damage they cause in chrysanthemums. The over-arching goal of this experiment was to determine which products (or product combinations) were the most effective at reducing RKNs in the soil, their infectivity in the roots of chrysanthemum and the damage they generate. The results from this experiment can help inform chrysanthemum growers to select biological nematicides, basic substances and biostimulants that are the best alternatives to steaming and chemical nematicides for combating RKN.

## Materials and methods

The experiment was conducted in the glasshouse at the Wageningen University and Research in Bleiswijk, the Netherlands from November 2019–January 2020. The plants were allowed to grow under the following conditions: 12/12 light/dark (i.e., short day cycle to induce flowering in chrysanthemum), average 402 PAR µmol•sec^-1^•m^-2^, with an average temperature of 18°C. The soil for the experiment was obtained from a chrysanthemum grower in November 2019. The growers noticed symptoms (both aboveground and belowground) in their chrysanthemum crop that matched typical damage caused by RKN. Before the experiment was initiated, the number of RKN in the soil was determined. On 12 November 2019, 10 samples weighing 50 g each of homogenized, untreated soil obtained from the growers were extracted for 3 d using the Baermann funnel method (Viglierchio and Schmitt, 1983; European and Mediterranean Plant Protection Organization, 2013). An additional five soil samples were taken and dried in an oven at 100 °C for 72 hr to determine the percentage dry weight of the soil so that the number of RKN per g dry weight soil could be calculated. After extraction, the number of RKN in each sample was determined using an inversion microscope (×40–200 magnification). The average number of RKNs across the 10 samples was: 3.7  ±  0.2 *Meloidogyne* spp. per g dry weight soil. The damage threshold for economic crop damage caused by *Meloidogyne* spp. in the soil can be as low as 1 egg per 100 cm^3^ soil or 0.5–2 J2 per gram soil (Greco and Di Vito, 2009). To account for artifact conditions in the glasshouse, the experiment was arranged in a randomized block design (i.e., each table was treated as a block, with two to three replicates of each treatment per table).

We tested three different reagents known to affect nematodes: one chemical (oxamyl) and one biological (garlic extract) and one basic substance (chitosan HCl; https://ec.europa.eu/food/plant/pesticides/eu-pesticides-database/public/?event=activesubstance.detail&language=EN&selectedID=1096 date queried: 10.06.2020) and five different biostimulants containing the following substances: microorganisms, plant oils, sea minerals, plant extracts and soldier fly waste (please see Table 1 for a detailed description of all the substances used). A control contaminated with RKN was included and received no other treatment. Another sterilized control (intended to mimic the effects of soil steaming) was included that was treated at 70°C for 1 hr to kill the majority of soil life, including the RKN (van der Wurff, 2010). Including the sterilized treatment allows for assessment of the efficacy of the treatments on reducing the number of RKN in the soil and the damage caused by RKN. All the reagents were applied individually to each plant and the chemical and biological nematicides, and the so-called basic substance, were applied in combination with the five biostimulant treatments and replicated 40 times (i.e., (3 × 5 = 15 combined treatments) + 10 individual treatments (control, negative control, 1 chemical nematicide, 1 biological nematicide, 1 basic substance, 5 biostimulants) = 25 total treatments × 40 replicates = 1000 experimental units).

**Table 1.** Experimental treatments.

Treatment name (product name)	Manufacturer	Active ingredient(s)	Description	Application instructions
Sterilized	NA	NA	Soil sterilized with an autoclave (1 hr, 70˚C)	NA
Control	NA	NA	Soil infested with *Meloidogyne* spp.; untreated	NA
Oxamyl^a^ (Vydate®)	Corteva	Oxamyl	Commercial chemical nematicide	0.04 g/liter soil/pot/plant; top layer
Garlic extract^b^	Anonymous	*Allium sativa* extract	Biological nematicide	0.04 g/liter soil/pot/plant; top layer
Chitosan HCl (DB Chitis 3.0)	De Broers	Chitin hydrochloride	Basic substance	50 ml/liter; 4 ml/plant
Microorganisms (Biovin)	Plant Health Cure	Microorganisms and micronutrients	Biostimulant/fertilizer	40 g/10-liter soil; 1-liter soil/pot/plant
Plant oils	Anonymous	Plant oils	Biostimulant	4 ml/liter; 4 ml/plant
Sea minerals^c^	Anonymous	Unprocessed sea minerals	Biostimulant/fertilizer	0.5 g/liter; 4 ml/plant; every 3 weeks
Plant extracts (Nemater)	Pireco	Plant extracts	Biostimulant	5 ml/liter; 5 ml/plant
Soldier fly waste (Flytilizer X)	Protix	Insect skins, frass, food fibers	Biostimulant	2 g/liter soil/pot/plant

Note: ^a^Chemical formula: C_7_H_13_N_3_O_3_^b^Liberated Allicin transformed polysulfides ^c^Contains: magnesium, calcium, sulphur, potassium, phosphorus, nitrogen, iron, boron, sodium, chloride and salt crystals. All of the reagents listed below were applied individually to the soil in which each plant grew and the chemical and biological nematicides, and the so-called basic substance, were applied in combination with the five biostimulant treatments (i.e., 3 × 5 = 15 combined treatments + 10 individual treatments = 25 total treatments).

Next, each 1-liter pot was prepared by adding the untreated (i.e., soil infected with RKN obtained from the grower) or sterilized soil. Between 19 and 21 November 2019, one chrysanthemum plant of the cultivar “Baltica” was planted in each pot. Each product was added to the designated pot according to the instructions from the manufacturer (Table 1). Plants were watered freely daily.

After 8 weeks of growth, the experiment was harvested between 15 and 17 January 2020. The aboveground portion of each plant was clipped and weighed. Thereafter, the belowground portion of each plant was carefully washed free from soil/peat block particles using tap water, lightly patted dry and weighed. The belowground portion of each plant was assigned a number on the root-knot index (RKI) (Bridge and Page, 1980). The RKI assigns a score to the level of damage caused by RKN, with a score of 0 = no root-knots and a score of 10 = roots completely covered in root-knots and the plant is dead or dying. All above- and belowground portions of the plants were then placed into paper bags and dried in an oven at 60°C for a minimum of 72 hr before the dry weight of each portion was taken.

A subset of pots (i.e., five plants from each treatment; *n* = 120) was randomly selected to assess the free-living and plant-parasitic nematode community in the soil. A 50 g wet weight soil sample from each pot was extracted using the Baermann funnel method and another subsample was dried to determine soil dry weight, as described above. This was done to determine the proportion of nematodes in the soil that belong to different feeding groups: plant-feeding, bacterial-feeding, fungal-feeding and omnivore-carnivore (Yeates et al., 1993). A healthy soil nematode community is important to support the health, resistance and resilience and nutrient cycling in the soil (Griffiths, 1994; Kulmatiski et al., 2014). Further, the amount of J2 *Meloidogyne* spp. was measured in the same soil samples, described above. The amount of J2 RKN in the soil indicates the potential infection risk for the following crop of chrysanthemum grown in the soils (van der Wurff, 2010).

The roots from the same subset of plants as the soil samples used for nematode extraction were placed into a mist chamber to extract the J2 RKN that hatched from the eggs attached to the roots (Teklu et al., 2013). The roots were cut into *c*. 1-cm pieces, the wet weight determined and then placed into a sieve with 150-µm net and the sieve was placed over a shallow container to catch the hatching J2 RKNs. The samples were placed into a mist chamber that sprayed warm water (*c*. 20 to 25 ˚C) over the roots for 15 min, followed by a 15 min pause in a continuous cycle. The roots remained in the mist chamber for 4 weeks (weekly checks occurred). Afterward, the water in the shallow containers was rinsed through a 20-µm sieve to catch the nematodes. The J2 RKN were then counted under an inversion microscope. The number of J2 RKN per gram wet weight roots was calculated.

Using the nematode data, we calculated several indices to determine the effect of the treatments on the functioning of the soil ecosystem. We used the Maturity Index (MI) to indicate the development of the soil from disturbed, labile nutrient-rich and highly unstructured to undisturbed, more recalcitrant energy channels and more structured based on the nematode community composition (Bongers, 1990, 1999). Nematode families are ranked from 1 to 5, ranging from r-strategists (c-p = 1) to extreme K-strategists (c-p = 5), with higher proportions of r-strategists and K-strategists indicating higher disturbed/polluted and undisturbed/unpolluted soil systems, respectively. Further, using rankings from the c-p scale, the Enrichment Index (EI) and the Structure Index (SI) were calculated to give an indication of how the treatments affected nutrient usage within the broader soil food web (i.e., the activity of detrital consumers) and the developmental trajectory of the soil food web into a more connected state (i.e., following disturbance), respectively (Ferris et al., 2001; Ferris and Bongers, 2006).

Each response variable (above- and belowground fresh and dry weight, RKI, number of *Meloidogyne* spp. in the soil and the roots, the total, total plant-feeding, bacterial-feeding, fungal-feeding and omnivore-carnivore nematodes, Structure, Maturity and Enrichment indices) was analyzed with a general linear mixed model. The treatment (i.e., 25 different control, sterilized, nematicide, basic substance, biostimulant and/or combinations thereof) was taken as a fixed factor and block (i.e., the table on which each sample plant grew) was taken as a random factor. The data were analyzed in R (R Core Team, 2019) with the packages lme4/lmerTest (Bates et al., 2015; Kuznetsova et al., 2017) and all data were either ln(x) or ln(x + 1) transformed as necessary to ensure homoscedasticity and normality; see footnotes after Table 2 for specifics. When significant effects were detected, data were subjected to post-hoc tests using the emmeans/multcomp packages in R (Hothorn et al., 2008; Lenth, 2019) with Tukey HSD adjustment for multiple comparisons.

**Table 2.** Results of statistical analyses.

Response variable	Sum of squares	Mean sum of squares	Df	*F*-value (*p*-value)
Aboveground fresh weight	11.6	0.5	24, 961	11.1 (<0.001)
Aboveground dry weight	12.7	0.5	24, 962	10.2 (<0.001)
Belowground fresh weight	18.9	1.0	24, 937	13.3 (<0.001)
Belowground dry weight	25.6	1.1	24, 828	15.2 (<0.001)
Root-knot index	59.3	2.5	24, 952	10.9 (<0.001)
Total nematodes	31.2	1.3	24, 93	1.9 (0.019)
*Meloidogyne spp.* (soil)	18.4	0.8	24, 100	2.2 (0.004)
*Meloidogyne spp.* (roots)	57.0	2.4	24, 97	2.8 (<0.001)
Total plant-feeding nematodes	37.9	1.6	24, 100	2.7 (<0.001)
Bacterial-feeding nematodes	33.2	1.4	24, 92	2.1 (0.008)
Fungal-feeding nematodes	13.1	0.6	24, 93	2.4 (0.001)
Carnivore-omnivore nematodes	1.3	0.1	24, 100	2.1 (0.007)
Maturity index	0.5	0.0	24, 95	2.1 (0.005)
Enrichment index	1.0	0.0	24, 97	2.9 (<0.001)
Structure index	53.4	2.3	24, 94	3.5 (<0.001)

Note: Data ln x or ln (*x* + 1) transformed before analysis. Aboveground fresh and dry weight *n* = 1,000; belowground fresh weight: *n* = 975; belowground dry weight: *n* = 866; Root-knot index: *n* = 990. Df = degrees of freedom, denominator degrees of freedom. All nematode variables: *n* = 120. The effects of the treatments on above- and belowground fresh and dry weight, the root-knot index (i.e., the level of damage caused to the chrysanthemums by the root-knot nematodes), the different nematode feeding groups, the number of *Meloidogyne* spp. extracted from the soil and the roots of the chrysanthemums and the nematode Enrichment, Maturity and Structure indices.

Finally, we used the Nematode Indicator Joint Analysis (NINJA) automated calculation system to obtain several visual metrics showing how the treatments affected colonizer-persister nematode composition and the food web in the soil (Sieriebriennikov et al., 2014). We plotted the colonizer-persister triangle to show how the treatments shifted the soil nematode communities to different states of stress, stability and enrichment. Further, we plotted the SI on the *x*-axis against the EI on the *y*-axis to give an indication of how the different treatments drove the soil food web into different states of stability (SI) and nutrient use strategy (EI) (Ferris and Bongers, 2006).

## Results

**Figure 1:**
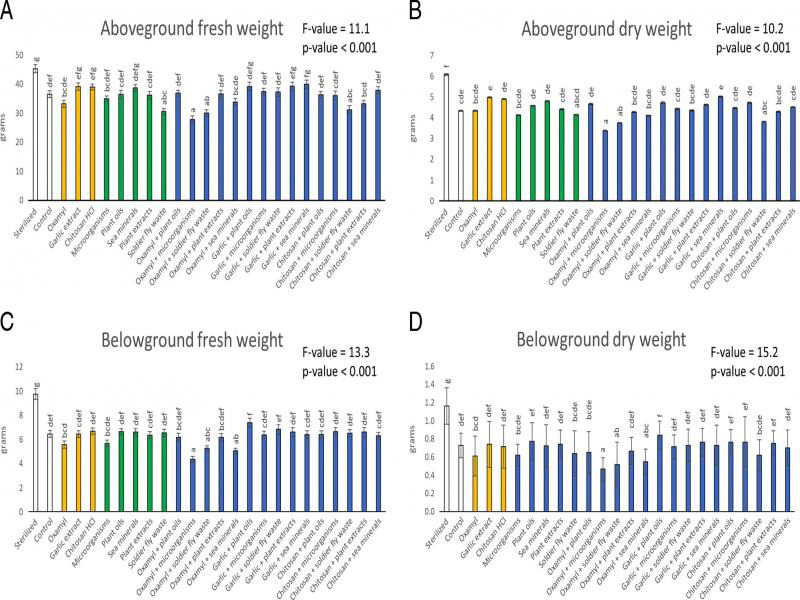
Aboveground fresh (A) and dry (B) and belowground fresh (C) and dry (D) weight of the chrysanthemums exposed to the different treatments at the end of the experiment. Within each panel, bars with different letters differ statistically significantly from one another (Tukey’s HSD p ≤ 0.05). Data shown are means ± SE.

**Figure 2:**
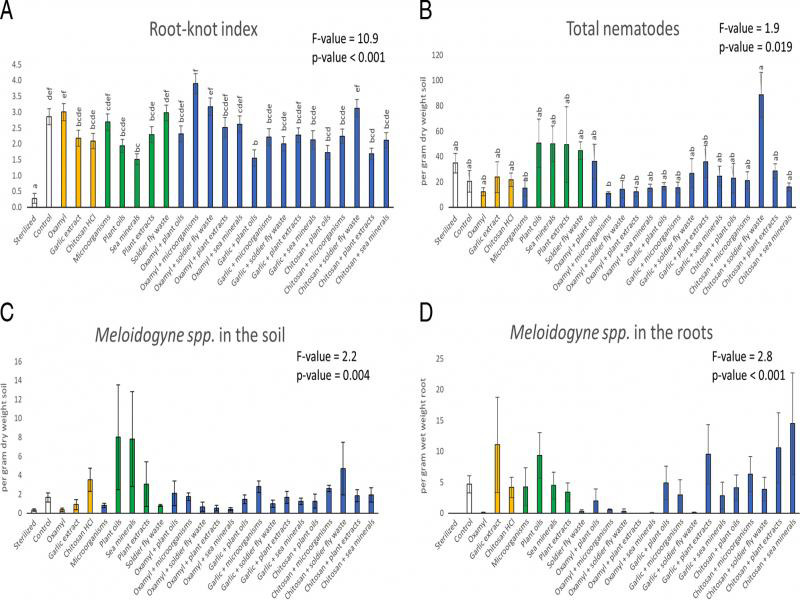
The root-knot index show the amount of damage caused by root-knot nematodes (*Meloidogyne* spp.) in the chrysanthemum roots: 0=no root-knots, 10=root entirely covered in knots and plant is dead or dying (A). Total nematodes (B) and *Meloidogyne* spp. in the ground (C) and *Meloidogyne* spp. extracted from the roots (D) of the chrysanthemums at the end of the experiment. Within each panel, bars with different letters differ statistically significantly from one another (Tukey’s HSD p ≤ 0.05); no letters indicate no statistically significant differences were detected. Data shown are means ± SE.

**Figure 3:**
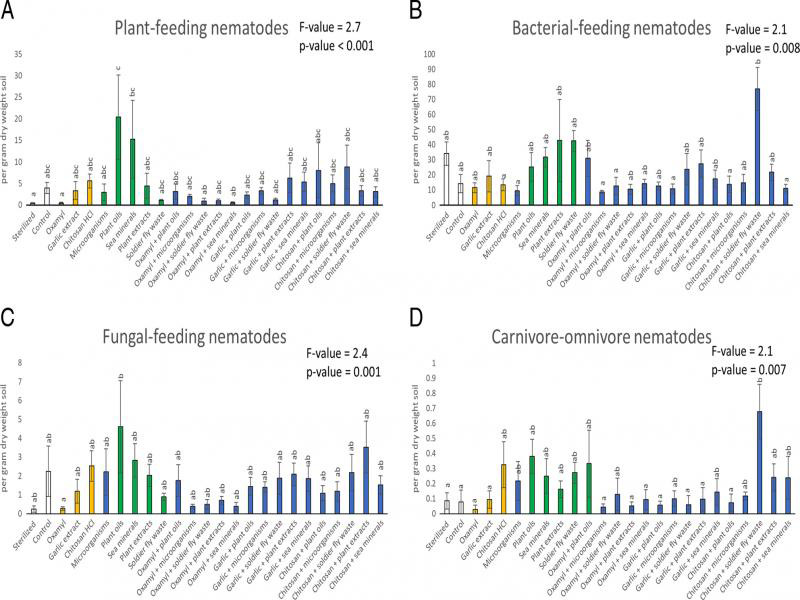
Total plant-feeding (A), bacterial-feeding (B), fungal-feeding (C) and carnivore-omnivore (D) nematodes that were extracted from soils of the chrysanthemums at the end of the experiment. Within each panel, bars with different letters differ statistically significantly from one another (Tukey’s HSD p ≤ 0.05). Data shown are means ± SE.

**Figure 4:**
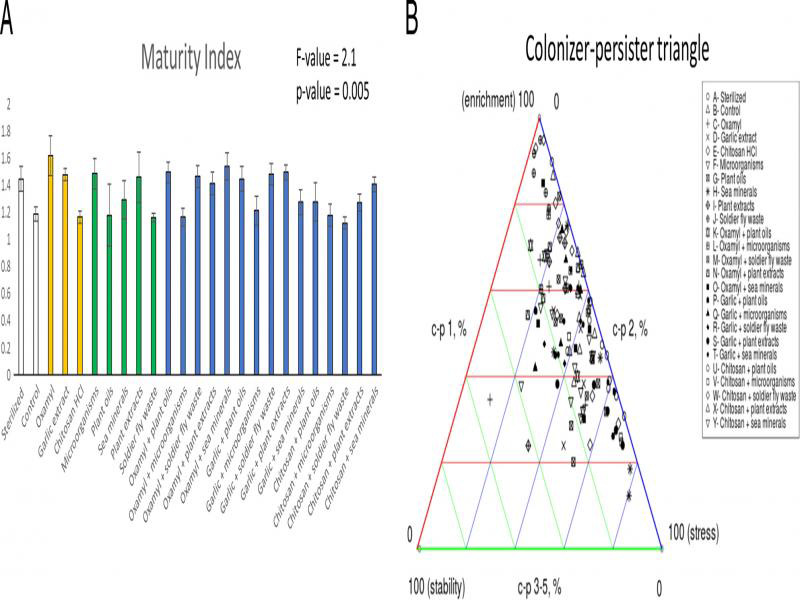
Effect of the treatments on the nematode Maturity Index (A) and the Colonizer-persister Triangle (B). Data shown are means ± SE.

**Figure 5:**
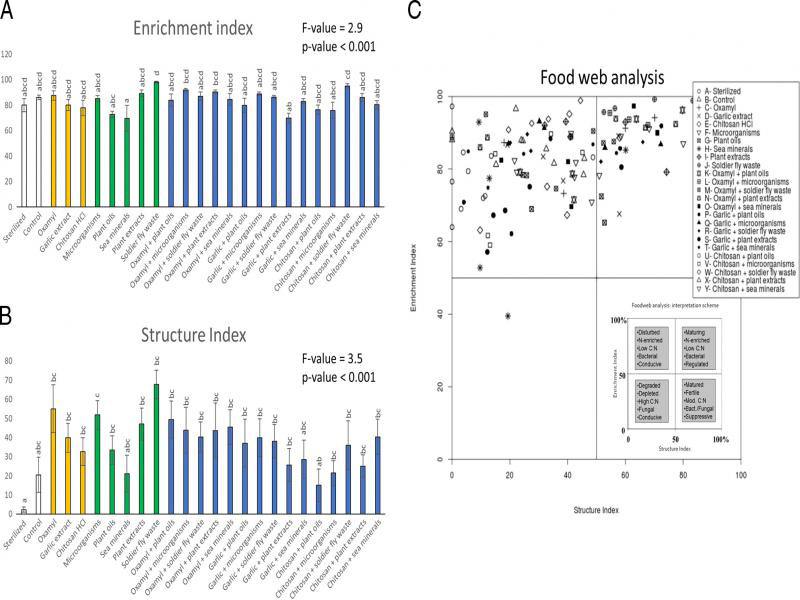
Effect of the treatments on the nematode Enrichment Index (A), Structure Index (B) and the food web analysis (including interpretation scheme inset) (C). Within A) and B), bars with different letters differ statistically significantly from one another (Tukey’s HSD p ≤ 0.05). Data shown are means ± SE.

All response variables were affected by the different treatments (Table 2 and Figures 1–5). However, post-hoc tests showed that not all variables differed significantly from the control treatment. Compared to the control, the aboveground fresh weight was 24% higher in the sterilized treatment and 16%, 24%, 18% and 15% lower in the soldier fly waste, oxamyl + microorganisms, oxamyl + soldier fly waste and chitosan HCl + soldier fly waste treatments, respectively (Figure 1A). Aboveground dry weight was 41% higher in the sterilized treatment and 22% and 13% lower in the oxamyl + microorganism and oxamyl + soldier fly waste treatments, respectively (Figure 1B). The belowground fresh weight was 51% higher in the sterilized treatment and 32% and 22% lower in the oxamyl + microorganism and the oxamyl + sea mineral treatments, respectively (Figure 1C). Belowground dry weight was 60% higher in the sterilized treatment and 36%, 29% and 25% lower in the oxamyl + microorganism, oxamyl + soldier fly waste and the oxamyl + sea mineral treatments, respectively (Figure 1D). The RKI was 90% lower in the sterilized treatment and *c*. 47% lower in both the sea mineral and the garlic extract + plant oil treatments (Figure 2A). Finally, the number of carnivore-omnivore nematodes was 756% higher in the chitosan HCl + soldier fly waste treatments (Figure 3D).

The MI was affected by the treatments, but post-hoc tests revealed that there were no true significant differences between treatments (Figure 4A). The EI was c. 28% higher in the soldier fly waste treatment than the plant oil, sea mineral and garlic + plant extracts treatments, respectively, but there were no differences between the control and any of the treatments (Figure 5A). The SI was the lowest in the sterilized treatment and was significantly different from all of the other treatments (c. 94% lower) except the control, sea mineral and the chitosan + plant oils treatments; the control treatment was not different from any treatment (Figure 5B). The colonizer-persister triangle (Figure 4B) showed that all soils were highly enriched and/or stressed, with no clear patterns emerging between treatments. The food web analysis diagram (Figure 5C) showed that (almost) all soils were highly enriched and the structure varied greatly, but again, with no distinct patterns emerging between treatments.

Although a number of the treatments did not differ significantly from the control statistically according to the post-hoc tests, there were some striking trends worth discussing. Compared to the control, the garlic extract, chitosan HCl and sea mineral treatments resulted in a *c*. 7% increase in aboveground fresh weight (Figure 1A) and a *c*. 14% increase in the aboveground dry weight (Figure 1B). Further, the garlic extract and the chitosan HCl treatments created a 24% and 27% reduction in the RKI, respectively, and combinations between these two reagents and several of the biostimulants caused a reduction in the RKI (Figure 2A). Finally, the total number of RKN in the soil was approximately 47% less in the garlic extract, microorganism and soldier fly waste treatments (Figure 2C). There was also a *c*. 138% increase in the average total number of nematodes in the soil with the separate addition of all of the biostimulants (with the exception of the microorganism treatment) (Figure 2B).

## Discussion

As expected, the sterilized treatment increased the overall above- and belowground weight of the chrysanthemums. In addition to directly killing pests and pathogens (van der Wurff, 2010), sterilization of soil causes a flush of nutrients, due to release from soil particles and the lysing of the cells of microorganisms and the death and decay of soil life in general (McNamara et al., 2007). This nutrient release often benefits the plants via increased growth. Sterilization also caused a reduction in the damage caused by the RKN as measured by the RKI, due to the direct death of the RKN (van der Wurff, 2010). Further, the sterilized soils had the lowest SI value, which indicates that soil communities in these soils were in the rudimentary stages of development (Ferris et al., 2001). This is intuitive because all soil organisms were killed and recolonization of soils into more developed states takes significant time (Bollen, 1974; Okada et al., 2004; Liu et al., 2006).

The use of the biological nematicide derived from garlic extract and the basic substance chitosan HCl (strong trends), as well as the biostimulants from sea minerals (statistically different from the control), had a positive effect against damage caused by RKNs. Specifically, the RKI was on average 47% lower in the sea mineral treatment and there was a trend towards a *c*. 25% reduction in the RKI with the addition of the garlic extract and the chitosan HCl treatments. These effects may have been caused by direct effects against the RKNs (Akhtar and Alam, 1993; Chen and Peng, 2019) or through indirect effects, such as an increase in plant resistance and resilience (Pylak et al., 2019) or changes to the soil microbiome. More specifically in relation to the soil microbiome, the observed positive effects of certain biostimulants on plant growth may have been caused by benefits to microorganisms due to increased nutrient availability (Buée et al., 2009) and through positive feedbacks, e.g., through root exudation favorable to symbiotic microorganisms, which in turn strengthen the plants and improve their growth (Philippot et al., 2013). However, combining garlic extract and chitosan HCl with certain biostimulants resulted in higher RKI values (albeit not significantly different from the control). This might be explained by complex interactions between active ingredients in the biostimulants, plant defense compounds and the rhizosphere community, which were beyond the scope of this study to qualify. Measurements on the secondary metabolites in the plants and rhizosphere microorganisms would be necessary to confirm if these effects were caused by changes to resistance due to, e.g., increased or decreased concentrations of defense compounds (Sato et al., 2019) and/or changes to the soil microbiome (Chen and Peng, 2019), respectively. If we could determine which product or combination of products created the most beneficial soil communities, then we could potentially steer the soil community in a way that improves plant resistance and resilience to RKNs.

In addition, it was unexpected that the application of oxamyl did not reduce the damage caused by the RKN, but it did reduce their presence in the soil and roots at the end of the experiment, albeit not statistically significant. However, similar mixed effects of oxamyl application have been seen in other studies (Bunt, 1975; Desaeger et al., 2004). Repeated application of oxamyl can lead to the evolution of a nematode population that is resistant to its effects (Glazer et al., 1997), but Yeates and Barker (1986) found no development of resistance), which may explain why the RKI was unaffected. Interestingly, oxamyl does not always kill nematodes directly (Ebone et al., 2019), but rather paralyzes them, with eventual recovery from paralysis and resumption of normal feeding activities after a few weeks (Wright and Womack, 1981). The resultant decrease in J2 RKNs in the soil and roots may have been the result of a hindered reproductive effort, due to paralysis of the RKN or the direct effects of oxamyl on the hatching of the RKNs (Evans and Wright, 1982; Woods et al., 1999). In essence, the nematodes may have recovered quickly enough from the paralysis caused by the application of oxamyl to damage the chrysanthemum, but were unable to successfully reproduce before the end of the 8-week growth period (Wright and Womack, 1981).

Several of the treatments caused a decrease in plant weight, e.g., the black soldier fly waste and various combinations between the oxamyl and some of the biostimulants. It is possible that these treatments caused a trade-off between growth and defense (Jones and Hartley, 1999), but this is not supported by the data, since no observed reduction in the RKI was seen in these treatment combinations. Instead, the reduction in plant weight in certain treatments may have resulted from the broad spectrum toxicity of oxamyl (Bunt, 1975; Bell et al., 2006), which perhaps negated the positive effects of certain biostimulants and may have inhibited recolonization of beneficial soil organisms (Roux-Michollet et al., 2008). On the other hand, the garlic extract, chitosan HCl and the sea mineral treatments resulted in plants with a *c*. 7% higher aboveground fresh weight. This is a modest increase but encouraging that the application of these products could help increase production levels in chrysanthemum.

Despite statistical insignificance, the amount of J2 RKNs in the soil was *c*. 47% lower in the garlic extract, microorganism and black soldier fly waste treatments. This suggests that the following chrysanthemum crop grown in these soils could experience less RKN damage. Repeated application of these products during the next growing cycle could strengthen these effects and should be explored further. Furthermore, the addition of nearly all the biostimulants resulted in a greater total number of nematodes in the soil. A greater number of nematodes in the soil is associated with faster nutrient cycling (Griffiths, 1994; Kulmatiski et al., 2014), which can benefit the following crop of chrysanthemums. Further, more nematodes in the soil are typically indicative of a healthier soil ecosystem (Bongers and Ferris, 1999). This means certain products may increase the resistance and resilience of the soil towards, not only RKN but potentially other pests and diseases. However, the colonizer-persister triangle and the food web analysis showed that, in general, all the soils were in early stages of development due to high disturbance rates, high nutrient enrichment, low C:N ratios and a bacteria-dominated energy channel (Ferris et al., 2001; Sieriebriennikov et al., 2014). This means that the ability of any of the treatments applied in this study to buffer against pests, diseases and other environmental stresses is likely limited.

The next step is to determine if these products have a stronger effect if they are applied over longer periods, that is to say, repeatedly administered for multiple chrysanthemum growth cycles. It is also important to investigate if these products yield similar results when added to different cultivars of chrysanthemum, since only one cultivar was used in this experiment and it is known that different varieties may respond differently due to inherent constitutional differences (Rohde, 1972; Giebel, 1974). If it is determined that chrysanthemum cultivars with specific characteristics react to these products differently, breeders would be able to select for these traits, alongside traits that also make the plants naturally resistant to RKN. In sum, a combined approach between the addition of biological pesticides, basic substances and biostimulants, alongside rigorous breeding programs, will help create more sustainable chrysanthemum cultivation.
